# A phase I study of intravenous and oral rucaparib in combination with chemotherapy in patients with advanced solid tumours

**DOI:** 10.1038/bjc.2017.36

**Published:** 2017-02-21

**Authors:** Richard H Wilson, TR Jeffry Evans, Mark R Middleton, L Rhoda Molife, James Spicer, Veronique Dieras, Patricia Roxburgh, Heidi Giordano, Sarah Jaw-Tsai, Sandra Goble, Ruth Plummer

**Affiliations:** 1 Centre for Cancer Research and Cell Biology, Queen's University Belfast, 97 Lisburn Road, Belfast BT9 7AE, UK; 2 Northern Ireland Cancer Center, Belfast City Hospital, 51 Lisburn Road, Belfast BT9 7AB, UK; 3 Beatson West of Scotland Cancer Centre, and Institute of Cancer Sciences, University of Glasgow, Garscube Estate, Switchback Road, Bearsden, Glasgow G61 1BD, UK; 4 National Institute for Health Research Biomedical Research Centre, Churchill Hospital, and Department of Oncology, University of Oxford, Old Road Campus Research Building, Roosevelt Drive, Oxford OX3 7DQ, UK; 5 Drug Development Unit, Royal Marsden Hospital/Institute of Cancer Research, Downs Road, Sutton, Surrey SM2 5PT, UK; 6 Division of Cancer Studies, King's College London, Guy's Hospital, Great Maze Pond, London SE1 9RT, UK; 7 Department of Medical Oncology, Institut Curie, 26, rue d'Ulm, Paris 75005 France; 8 Clovis Oncology, Inc., Boulder, 5500 Flatiron Parkway, Boulder, CO 80301, USA; 9 Northern Centre for Cancer Care, Freeman Hospital, Freeman Road, High Heaton, Newcastle Upon Tyne NE7 7DN, UK; 10 Northern Institute for Cancer Research, Newcastle University, Paul O'Gorman Building, Framlington Place, Newcastle Upon Tyne NE2 4HH, UK

**Keywords:** rucaparib, carboplatin, PARP inhibitor, pharmacokinetics and pharmacodynamics, breast cancer, ovarian cancer, pancreatic cancer, BRCA1, BRCA2

## Abstract

**Background:**

This study evaluated safety, pharmacokinetics, and clinical activity of intravenous and oral rucaparib, a poly(ADP-ribose) polymerase inhibitor, combined with chemotherapy in patients with advanced solid tumours.

**Methods:**

Initially, patients received escalating doses of intravenous rucaparib combined with carboplatin, carboplatin/paclitaxel, cisplatin/pemetrexed, or epirubicin/cyclophosphamide. Subsequently, the study was amended to focus on oral rucaparib (once daily, days 1–14) combined with carboplatin (day 1) in 21-day cycles. Dose-limiting toxicities (DLTs) were assessed in cycle 1 and safety in all cycles.

**Results:**

Eighty-five patients were enrolled (22 breast, 15 ovarian/peritoneal, and 48 other primary cancers), with a median of three prior therapies (range, 1–7). Neutropenia (27.1%) and thrombocytopenia (18.8%) were the most common grade ⩾3 toxicities across combinations and were DLTs with the oral rucaparib/carboplatin combination. Maximum tolerated dose for the combination was 240 mg per day oral rucaparib and carboplatin area under the curve 5 mg ml^−1^ min^−1^. Oral rucaparib demonstrated dose-proportional kinetics, a long half-life (≈17 h), and good bioavailability (36%). Pharmacokinetics were unchanged by carboplatin coadministration. The rucaparib/carboplatin combination had radiologic antitumour activity, primarily in *BRCA1-* or *BRCA2*-mutated breast and ovarian/peritoneal cancers.

**Conclusions:**

Oral rucaparib can be safely combined with a clinically relevant dose of carboplatin in patients with advanced solid tumours (Trial registration ID: NCT01009190).

The poly (ADP-ribose) polymerase (PARP) family includes enzymes that are involved in the repair of single-strand breaks, a common type of DNA damage, thus preventing the formation of DNA double-strand breaks (Schreiber *et al*, 2006). The DNA double-strand breaks can be repaired by a separate process known as homologous recombination, mediated by *BRCA1* and *BRCA2* (Moynahan *et al*, 1999, 2001; Venkitaraman, 2002). Tumours harbouring a *BRCA* mutation or other defect in homologous recombination repair are sensitive to PARP inhibitors, because cells accumulate unrepaired single-strand breaks that are converted to double-strand breaks that cannot be repaired and therefore result in cell death (Bryant *et al*, 2005; Farmer *et al*, 2005; Helleday *et al*, 2007, 2008; Ashworth, 2008). Furthermore, studies have demonstrated that PARP inhibition can interfere with the alternative nonhomologous end-joining DNA repair pathway that is upregulated in homologous recombination-deficient cells (Helleday, 2011; Ceccaldi *et al*, 2015; Konstantinopoulos *et al*, 2015; Mateos-Gomez *et al*, 2015). The PARP inhibitors can also result in trapping of PARP-1 and PARP-2 at the site of the DNA break, resulting in obstructed replication forks that require functional homologous recombination for repair (Helleday, 2011; Murai *et al*, 2012; O'Connor, 2015).

Consistent with the role of PARPs in DNA repair, inhibiting PARP has been shown to increase the potency of DNA-damaging agents, such as chemotherapy and radiotherapy (Calabrese *et al*, 2004; Donawho *et al*, 2007; Thomas *et al*, 2007; Ihnen *et al*, 2013). Preclinical data suggest that a PARP inhibitor in combination with carboplatin or cisplatin has enhanced efficacy over either agent individually in *BRCA*-mutated tumours (Evers *et al*, 2008; Drew *et al*, 2011; Clark *et al*, 2012). The synergistic effect of these combinations may be the result of an increase in DNA damage (e.g., intrastrand crosslinks) induced by platinum-based chemotherapies that requires repair through PARP-dependent pathways.

Rucaparib (formerly known as AG-014699 and PF-01367338) is a potent small-molecule inhibitor of PARP-1, PARP-2, and PARP-3 that is being developed for the treatment of ovarian cancer and other tumour types associated with homologous recombination deficiency (HRD), including *BRCA1* and *BRCA2* mutations (Thomas *et al*, 2007; Drew *et al*, 2011; Swisher *et al*, 2017). In a phase II study in patients with advanced ovarian or breast cancer associated with a germline *BRCA1/2* mutation, continuous dosing of single-agent oral rucaparib led to a higher rate of response than intermittent intravenous (i.v.) dosing (response rate, 18% *vs* 2%) (Drew *et al*, 2016). A subsequent phase I–II dose-escalation study established the recommended phase II dose of single-agent oral rucaparib as 600 mg twice daily (Kristeleit *et al*, 2014) and demonstrated the clinical activity and manageable safety profile of rucaparib in patients with advanced solid tumours, including *BRCA*-mutated ovarian and breast cancers (Kristeleit *et al*, 2014; Shapira-Frommer *et al*, 2015). In phase I and II studies, the combination of i.v. rucaparib and the DNA-alkylating agent temozolomide was active in patients with advanced solid tumours and resulted in increased activity compared with historical data of single-agent temozolomide in patients with metastatic melanoma (Plummer *et al*, 2008, 2013).

This phase I dose-escalation study evaluated rucaparib in combination with several standard chemotherapeutic regimens in patients with advanced solid tumours, independent of *BRCA* status. The study initially explored an i.v. formulation of rucaparib; however, during the conduct of this study, an oral formulation of rucaparib was developed that could be administered for a longer duration, and the study was amended to evaluate the oral bioavailability of this new formulation. Once bioavailability was established, the study was amended to evaluate oral rucaparib in combination with carboplatin. Here, we report final results from all patients enrolled in the study, with a focus on those who received oral rucaparib in combination with carboplatin.

## Materials and methods

### Study design

This study was an open-label, multicentre, dose-escalating phase I study of rucaparib administered in combination with one of four different standard chemotherapeutic regimens (NCT01009190). Eligible patients ⩾18 years of age had a histologically or cytologically confirmed advanced solid tumour, an Eastern Cooperative Oncology Group (ECOG) Performance Status of 0 or 1, life expectancy of ⩾12 weeks, and adequate bone marrow, liver, and renal function. All *BRCA* testing was done locally and was not verified by the sponsor. The primary objective was to assess safety and tolerability and estimate the maximum tolerated dose (MTD) and/or select the recommended phase II dose of rucaparib in combination with chemotherapy. Secondary objectives were to characterise the pharmacokinetics (PK) and assess the antitumour activity of rucaparib when combined with chemotherapy.

The study was approved by the Research Ethics Committee for all participating institutions and conducted in accordance with the Declaration of Helsinki and the Good Clinical Practice Guidelines of the International Conference on Harmonisation. Patients gave written informed consent before undergoing any study-related procedures.

### Treatments

**Figure 1:**
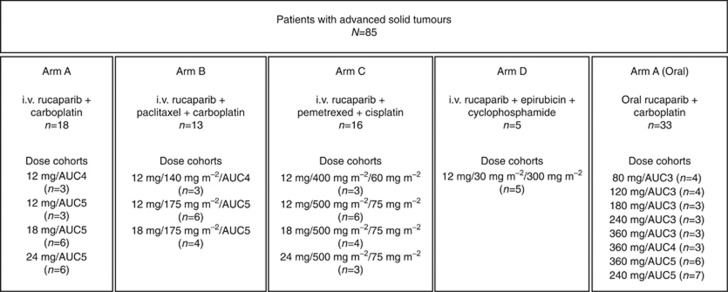
**Study treatment arms.**

Patients received escalating doses of i.v. rucaparib (days 1–3) with standard doses of chemotherapeutic regimens. Initial starting doses for each chemotherapy were as follows: arm A, rucaparib (24 mg)+carboplatin (area under the curve 4 mg min ml^−1^ (AUC4)); arm B, rucaparib (24 mg)+carboplatin (AUC4)+paclitaxel (140 mg m^−2^); arm C, rucaparib (24 mg)+cisplatin (60 mg m^−2^)+pemetrexed (400 mg m^−2^); and arm D, rucaparib (12 mg)+epirubicin (30 mg m^−2^)+cyclophosphamide (300 mg m^−2^) (Figure 1). Initially, patients received chemotherapy (day 1) and i.v. rucaparib (days 1–3) in 21-day treatment cycles. However, during the conduct of the study, an oral formulation of rucaparib was developed and introduced under a protocol amendment, with an additional secondary objective to determine its absolute oral bioavailability. Subsequently, the i.v. rucaparib arms were discontinued and three of the chemotherapy arms (B, C, and D) were closed to further enrolment. Thereafter, all enrolled patients received oral rucaparib in combination with i.v. carboplatin (arm A, oral rucaparib) (Figure 1).

Treatment with rucaparib in combination with chemotherapy was continued until progression, unacceptable toxicity, patient's withdrawal of consent, or as deemed appropriate by the judgement of the treating physician (whichever came first).

#### Oral rucaparib in combination with carboplatin

Patients received lead-in doses of i.v. and oral rucaparib on days −10 and −5, respectively, followed by carboplatin (AUC3, 4, or 5) on day 1 and oral rucaparib on days 1–14 of every 21-day treatment cycle. The i.v. lead-in dose of rucaparib was discontinued once sufficient bioavailability data for oral rucaparib were available. The starting dose of 80 mg oral rucaparib was based on the safety established with up to 24 mg m^−2^ i.v. rucaparib in combination with chemotherapy and an oral bioavailability of 36%. The protocol prespecified dose cohorts of 80, 120, and 180 mg oral rucaparib, after which rucaparib was to be escalated in 50% increments. Additional doses of 240 and 360 mg were subsequently evaluated. Similarly, carboplatin dosing began at AUC3, with a plan to escalate to AUC5 once the MTD of rucaparib was established; however, escalation was modified to evaluate AUC4 carboplatin before escalating to AUC5. Patients discontinuing carboplatin could continue to receive oral rucaparib.

### Safety and efficacy assessments

Safety assessments included collection of adverse events (AEs) and serious AEs (defined by National Cancer Institute Common Terminology Criteria for Adverse Events version 3.0; Pedersen *et al*, 2013), as well as vital signs, physical examination, 12-lead electrocardiogram, laboratory assessments, and verification of concurrent medications. Safety variables and demographic data were presented descriptively. The safety analysis set included all enrolled patients who started treatment.

Dose-limiting toxicities (DLTs) were defined as any of the following occurring during cycle 1: grade 4 neutropenia lasting ⩾7 days; febrile neutropenia; grade 3 thrombocytopenia lasting ⩾7 days with bleeding or grade 4 thrombocytopenia lasting >3 days; grade ⩾3 toxicity despite the use of adequate/maximal medical interventions and/or prophylaxis as dictated by local institutional clinical practices or the judgement of the investigator; grade 2 neurotoxicity that did not recover to grade ⩽1 within 2 weeks of planned dose; toxicities that resulted in a delay of >14 days in initiation of cycle 2 dosing; or toxicities that resulted in failure to deliver ⩾80% of the assigned oral rucaparib doses.

Initially, at least three patients were treated at each dose level. If no DLT was observed, the dose was escalated for the next cohort of three patients. If a DLT was observed in one of the three patients, three additional patients were enrolled and treated at the same dose level. If no further DLT was observed, the next dose level was opened. Dose escalation continued until DLTs were observed in at least two of the three to six patients treated at that dose level; this dose was then considered above the MTD and further dose escalations were stopped. When a dose was concluded to be above the MTD, the preceding lower dose was declared the MTD, but only if six patients had already been treated at this lower dose. Otherwise, three additional patients were treated at this lower dose, and if none or one of those patients had a DLT, this lower dose was declared the MTD.

All patients who received at least one dose of study medication were evaluable for toxicity. Patients were considered nonevaluable for DLT assessments if they missed the rucaparib lead-in doses, had administration of <80% of the planned cycle 1 doses of rucaparib, and/or had administration of <80% of the planned cycle 1 doses of chemotherapy for that dose level (provided that the reduction did not result from toxicity).

Antitumour activity was assessed by the investigators according to Response Evaluation Criteria in Solid Tumor version 1.1 (RECIST) (Eisenhauer *et al*, 2009) through radiological tumour assessments performed every two cycles and/or at the end of treatment, whichever occurred first.

### Pharmacokinetics

On cycle 1 days −10, −5, 1, and 14, plasma samples were obtained from all patients before rucaparib dosing and at 15 and 30 min, and at 1, 1.5, 2.5, 4, 6, 10, and 24 h after the start of the rucaparib administration (i.v. infusion (day −10) or oral (days −5, 1, and 14)). On cycle 1 days −10 and −5, samples were also obtained 48 h after the start of rucaparib administration. Rucaparib concentration was determined using liquid chromatography with tandem mass spectrometry. The PK assay was validated by York Bioanalytical Solutions (York, UK) in accordance with the US Food and Drug Administration's Bioanalytical Method Validation Guidance for Industry and Crystal City III Conference (US Food and Drug Administration, 2001; Viswanathan *et al*, 2007; Fast *et al*, 2009). The PK concentration analysis population was defined as all treated patients who had at least one concentration measurement in at least one treatment period (cycle 1). The PK parameter analysis population was defined as all treated patients who had at least one of the PK parameters of interest in at least one treatment period (cycle 1).

Standard plasma PK parameters for rucaparib were estimated using noncompartmental methods and included: maximum plasma drug concentration (C_max_); area under the plasma concentration time curve from time 0 to the last sampling time with measurable values (AUC_0–*t*_) and from time 0 to 24 h (AUC_0–24_); and half-life (*t*_1/2_). Plasma clearance or apparent plasma clearance and steady-state volume of distribution were calculated for rucaparib. The oral bioavailability of rucaparib was calculated as the ratio of dose-normalised AUC_0–*t*_ or AUC_inf_ (data permitting) determined using oral rucaparib PK data collected on cycle 1 day −5 to that determined using the i.v. PK data collected on cycle 1 day −10. Pharmacokinetic parameters were summarised with the geometric mean and the coefficient of variation (CV). The ratio of C_max_ and AUC_0–24_ of rucaparib administered in combination with chemotherapies to those of rucaparib alone was calculated and summarised, with mean and CV% to evaluate the effect of chemotherapies on the PK of rucaparib for both the i.v. dose (day −10) and oral dose (day −5). Additional PK parameters examined and analyses performed are described in the Supplementary Methods.

## Results

### Patients and treatments

Eighty-five patients were enrolled at seven sites in the United Kingdom and France (Table 1). Median age was 55 years (range, 20–76 years), approximately two-thirds of patients were female, and all had an ECOG Performance Status of 0 or 1. Of the 85 patients, 22 (25.9%) had breast cancer, 15 (17.6%) had ovarian/peritoneal cancer, 8 (9.4%) had lung cancer, 4 (4.7%) each had pancreatic or rectal cancer, 31 (36.5%) had other primary cancers, and 1 (1.2%) patient had a carcinoma of unknown primary origin. The *BRCA* test results were unavailable for most patients (78.8% and 81.2% of patients did not undergo *BRCA1* or *BRCA2* testing, respectively). The median number of prior anticancer therapies was three (range, 1–7).

All patients had discontinued the study as of 2 April 2014. The median number of cycles initiated was 5 (range, 1–7) in arm A (i.v. rucaparib; *n*=18), 4 (range, 1–31) in arm A (oral rucaparib; *n*=33), 4 (range, 1–6) in arm B (*n*=13), 4 (range, 1–7) in arm C (*n*=16), and 1 (range, 1–4) in arm D (*n*=5). Eight of 18 (44.4%) patients from arm A (i.v. rucaparib) and 17 of 33 (51.5%) patients from arm A (oral rucaparib) had one or more delays (⩾1 week) between treatment cycles. These occurred between cycles 1 and 2 in 1 (5.6%) patient in arm A (i.v. rucaparib) and 9 (27.3%) patients in arm A (oral rucaparib). Four of 18 (22.2%) patients from arm A (i.v. rucaparib) had a dose reduction of carboplatin; 8 of 33 (24.2%) patients from arm A (oral rucaparib) had a dose reduction, including 6 (21.2%) of carboplatin, 1 (3.0%) of rucaparib, and 1 (3.0%) of both drugs. Across all cohorts, 76 (89.4%) discontinued treatment with the following as the primary reason: objective progression or relapse (42 patients, 55.3%), AE (14 patients, 18.4%), global deterioration of health status (8 patients, 10.5%), no longer willing to participate in the study (5 patients, 6.6%), and other (7 patients, 9.2%). Specifically, in arm A (oral rucaparib; *n*=33) patients discontinued treatment primarily because of objective progression or relapse (18 patients, 54.5%), global deterioration of health status (5 patients, 15.2%), AE (3 patients, 9.1%), no longer willing to participate in the study (2 patients, 6.1%), and other (5 patients, 15.2%).

### Dose-limiting toxicity and maximum tolerated dose

In arm A (oral rucaparib), no patients experienced a DLT at doses up to 360 mg rucaparib in combination with AUC4 carboplatin. The first three patients who received 360 mg rucaparib in combination with AUC5 carboplatin experienced dose interruptions associated with neutropenia, although these were grade ⩽3 and did not meet the protocol-specified definition of a DLT. Therefore, a decision was made to enrol an additional three patients to further evaluate the safety and tolerability of this dose combination. Dose-limiting toxicities of grade 4 thrombocytopenia and grade 4 neutropenia were observed in the first two patients treated, and thus the third additional patient was not enrolled. The rucaparib dose was deescalated to 240 mg and three patients were treated. One patient experienced a DLT (grade 4 thrombocytopenia), and thus an additional three patients were enrolled. None of these three additional patients had a DLT. Therefore, AUC5 carboplatin and 240 mg once daily (q.d.) oral rucaparib was declared the MTD.

Dose-limiting toxicities were also reported in one patient in arm B who was receiving 36 mg rucaparib, 306 mg paclitaxel, and 610 mg carboplatin (grade 3 diarrhoea and grade 3 nausea) and in one patient in arm C who was receiving 12 mg rucaparib, 900 mg pemetrexed, and 135 mg cisplatin (grade 3 fatigue, grade 4 leukopenia, and grade 4 neutropenia). Because of a decision to discontinue recruitment to the i.v. rucaparib arms during the study, no MTD was determined for arms A (i.v. rucaparib), B, C, and D that evaluated i.v. rucaparib in combination with chemotherapy.

### Adverse events

All except one patient experienced an AE during the study. Adverse events occurring in >20% of patients in any treatment group are summarised in Table 2. Across the treatment arms, AEs were generally grade 1 or 2 in severity. The most common AEs (with ⩾30% incidence in all patients) across groups were gastrointestinal events (i.e., nausea, constipation, vomiting, and diarrhoea), fatigue, and events related to myelosuppression (i.e., anaemia, neutropenia, and thrombocytopenia). Grade ⩾3 AEs were reported in 64 of 85 patients (75.3%), the most frequent of which were neutropenia (23 patients, 27.1%), thrombocytopenia (16 patients, 18.8%), fatigue (11 patients, 12.9%), anaemia (10 patients, 11.8%), nausea (6 patients, 7.1%), and the following in 5 patients (5.9%) each: vomiting, *γ*-glutamyltransferase increased, and dyspnoea. Myelosuppression was managed through transfusion or supportive medication when necessary; one patient (in arm C) received granulocyte colony-stimulating factor in response to grade 2 neutropenia. Treatment-related AEs, as assessed by investigators, were reported in 94.1% of patients (Supplementary Table S1).

Across treatment arms, 22 patients (25.9%) discontinued treatment because of AEs that included neutropenia (3 patients, 3.5%), thrombocytopenia (2 patients, 2.4%), and platinum hypersensitivity (2 patients, 2.4%). While on study, 6 patients (7.1%) died of disease progression that was assessed as unrelated to study drug.

### Pharmacokinetics

**Figure 2:**
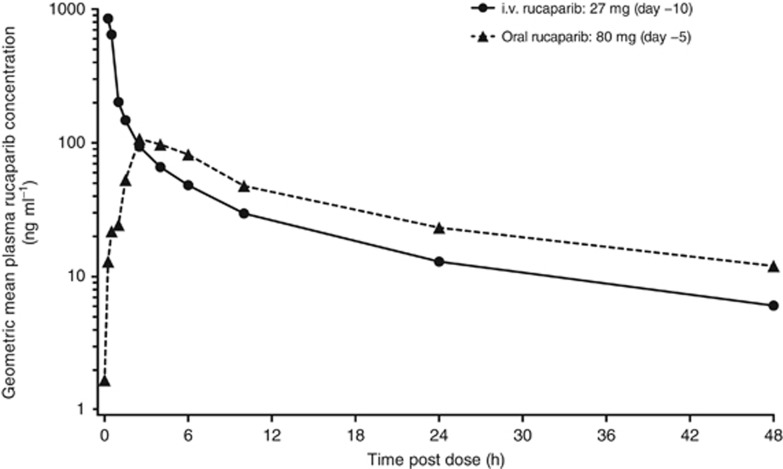
**Plasma rucaparib concentration–time profile following i.v. or oral administration.** Graph shows the geometric mean plasma concentration of rucaparib in patients in arm A (oral rucaparib) (*n*=4) who received i.v. and oral doses on days −10 and −5, respectively.

Rucaparib exposure increased approximately dose proportionally when given orally or intravenously (Supplementary Tables S2–S5). On study day 14, the steady-state AUC_0–24_ increased by an average of 61%, with no change in *t*_1/2_ compared with a single dose. Regardless of the administration route, the dose-independent *t*_1/2_ was ≈17 h as observed in patients from arm A (oral rucaparib) who received 27 mg i.v. rucaparib on day −10 and 80 mg oral rucaparib on day −5 (Figure 2). Rucaparib demonstrated good absorption, with a dose-independent mean oral bioavailability of 36% in the fasted state. Rucaparib oral PK was not affected by coadministration of AUC3 to AUC5 carboplatin (Table 3). No apparent impact on i.v. rucaparib PK was observed with coadministration of carboplatin+paclitaxel, cisplatin+pemetrexed, or epirubicin+cyclophosphamide (Table 3).

### Tumour response

Tumour response data were available for 77 of 85 patients. Across all cohorts, 1 patient (1.2%) with breast cancer achieved a confirmed complete response (CR) and 9 patients (10.6%) achieved a partial response (PR) that was confirmed in 7 patients (Table 4). Forty-three patients (50.6%) achieved stable disease (SD). Among patients with available data, across all cohorts, the disease control rate (CR, PR, or SD for ⩾12 weeks) was 68.8%. Three of 33 patients (9.1%) receiving oral rucaparib in combination with carboplatin had a confirmed PR. These included a patient with ovarian/peritoneal cancer (*BRCA1* mutation, *BRCA2* wild type) who had a PR for 7 months, a second patient with ovarian/peritoneal cancer (*BRCA1* and *BRCA2* wild type) who had a PR for 5 months, and a patient with breast cancer (*BRCA1* mutation, *BRCA2* not tested) who had a PR for 3 months. None of these patients had received prior PARP inhibitor therapy.

## Discussion

This phase I study demonstrated that oral rucaparib can be safely combined with a clinically relevant dose of carboplatin in patients with an advanced solid tumour. The MTD and recommended dose for the combination is 240 mg q.d. rucaparib on days 1–14 with AUC5 carboplatin on day 1 in 21-day cycles.

Oral rucaparib demonstrated dose-proportional kinetics and a long *t*_1/2_ (≈17 h) with good oral bioavailability (36%) independent of dose, all of which are desirable characteristics for an oral agent. These PK findings with oral rucaparib are consistent with results from the phase I portion of an ongoing phase I–II study evaluating single-agent oral rucaparib in patients with advanced solid tumours (Kristeleit *et al*, 2014). Pharmacokinetic exposure to oral rucaparib was not changed by carboplatin coadministration. When used in combination with AUC5 carboplatin, the i.v. rucaparib doses of 12, 18, and 24 mg evaluated were approximately equivalent to oral rucaparib doses of 33, 50, and 67 mg, respectively, representing 14%, 21%, and 28% of the MTD of 240 mg oral rucaparib.

As anticipated with the concurrent administration of chemotherapeutic agents with rucaparib, myelosuppression (anaemia, neutropenia, and thrombocytopenia) was commonly reported. Myelosuppression is a known toxicity associated with a high dose of PARP inhibitors and is also often observed with many chemotherapy regimens (Sandhu *et al*, 2013; Plummer *et al*, 2014; Kaufman *et al*, 2015; Shapira-Frommer *et al*, 2015). In our study, thrombocytopenia and neutropenia were DLTs and also the most common severe toxicities reported across all treatment arms. Myelosuppression was also observed in 54% of patients treated with the combination of i.v. rucaparib and temozolomide in another study (Plummer *et al*, 2013). The combination of the PARP inhibitor olaparib with chemotherapy, including carboplatin, has also been associated with substantial hematologic toxicity in phase I studies of patients with an advanced solid tumour (Khan *et al*, 2011; Rajan *et al*, 2012; Samol *et al*, 2012; Dent *et al*, 2013; Lee *et al*, 2014a).

Rucaparib exhibited clinical activity in combination with chemotherapy in this study of heavily pretreated patients with advanced malignancies. More than two-thirds of all patients in the study had stable disease or better, and this is notable because patients were not preselected for HRD (only 10 of 85 patients were known to harbour a *BRCA* mutation). Of patients who received oral rucaparib and carboplatin, 63.6% achieved disease control for ⩾12 weeks, and PRs were reported in 3 patients with ovarian or breast cancer, 2 of whom had a known *BRCA* mutation. The benefits of inhibiting PARP in *BRCA*-mutated ovarian or breast cancer are well documented (Fong *et al*, 2009; Audeh *et al*, 2010; Tutt *et al*, 2010; Kaye *et al*, 2012; Sandhu *et al*, 2013; Lee *et al*, 2014b). As a single agent, oral rucaparib has demonstrated activity against *BRCA*-mutated ovarian and breast cancer (Shapira-Frommer *et al*, 2015; Drew *et al*, 2016; Swisher *et al*, 2017) and is currently being evaluated in phase III studies as both maintenance and treatment therapy for patients with relapsed, high-grade ovarian cancer (ARIEL3 (NCT01968213) and ARIEL4 (NCT02855944)).

When used in combination with carboplatin, the dose of rucaparib (240 mg q.d.) is lower than the MTD of single-agent rucaparib (600 mg twice daily). The lower dose of rucaparib in the combination fits with prior studies demonstrating a synergistic effect with combinations of PARP inhibitors and DNA-damaging agents at lower doses (Calabrese *et al*, 2004; Donawho *et al*, 2007; Thomas *et al*, 2007; Ihnen *et al*, 2013). Given that platinum-based chemotherapies increase the burden of DNA damage in cells, the addition of PARP inhibition increases the cytotoxicity of these agents by preventing repair of damaged DNA. Although PARP inhibitors in the single-agent setting have demonstrated benefits in DNA-repair-deficient cancers (e.g., *BRCA*-mutated ovarian cancer), combinations such as rucaparib and carboplatin may be useful in a wider population, including in cancers with and without homologous repair deficiency.

In conclusion, this study demonstrated that oral rucaparib can be safely combined with a clinically relevant dose of carboplatin; however, neutropenia and thrombocytopenia were commonly observed with the rucaparib/carboplatin combination. All patients who receive rucaparib in combination with carboplatin should be monitored carefully for myelosuppression. The oral and i.v. PK profile of rucaparib was not affected by coadministration of the chemotherapeutic agents that were investigated in this study. Three heavily pretreated patients who received oral rucaparib and carboplatin had a clinical response; further work investigating this combination may be warranted.

## Figures and Tables

**Table 1:** Patient demographics and disease characteristics

**Variable**	**Arm A, i.v. rucaparib+carboplatin (*n*=18)**	**Arm B, i.v. rucaparib+carboplatin/paclitaxel (*n*=13)**	**Arm C, i.v. rucaparib+cisplatin/pemetrexed (*n*=16)**	**Arm D, i.v. rucaparib+epirubicin/ cyclophosphamide (*n*=5)**	**Arm A, oral rucaparib+carboplatin (*n*=33)**	**All patients (*N*=85)**
Median age (range), years	51.5 (23–68)	61.0 (39–69)	50.5 (32–68)	41.0 (32–53)	61.0 (20–76)	55.0 (20–76)
Gender, *n* (%)						
Female	11 (61.1)	7 (53.8)	10 (62.5)	4 (80.0)	23 (69.7)	55 (64.7)
Male	7 (38.9)	6 (46.2)	6 (37.5)	1 (20.0)	10 (30.3)	30 (35.3)
Race, *n* (%)						
Asian	0	0	1 (6.3)	0	0	1 (1.2)
Black	1 (5.6)	1 (7.7)	0	0	0	2 (2.4)
White	17 (94.4)	12 (92.3)	15 (93.8)	5 (100.0)	33 (100.0)	82 (96.5)
ECOG PS, *n* (%)						
0	7 (38.9)	6 (46.2)	6 (37.5)	5 (100.0)	16 (48.5)	40 (47.1)
1	11 (61.1)	7 (53.8)	10 (62.5)	0	17 (51.5)	45 (52.9)
Primary cancer diagnosis, *n* (%)						
Breast	7 (38.9)	1 (7.7)	4 (25.0)	4 (80.0)	6 (18.2)	22 (25.9)
Ovarian/peritoneal	2 (11.1)	1 (7.7)	2 (12.5)	0	10 (30.3)	15 (17.6)
Lung	1 (5.6)	2 (15.4)	1 (6.3)	1 (20.0)	3 (9.1)	8 (9.4)
Pancreas	0	0	2 (12.5)	0	2 (6.1)	4 (4.7)
Rectal	3 (16.7)	0	0	0	1 (3.0)	4 (4.7)
Unknown primary	1 (5.6)	0	0	0	0	1 (1.2)
Other^a^	4 (22.2)	9 (69.2)	7 (43.8)	0	11 (33.3)	31 (36.5)
*BRCA1* test results, *n* (%)						
Positive	2 (11.1)	1 (7.7)	2 (12.5)	0	2 (6.1)	7 (8.2)
Negative	3 (16.7)	0	0	3 (60.0)	3 (9.1)	9 (10.6)
Unknown	0	0	0	0	2 (6.1)	2 (2.4)
Not tested	13 (72.2)	12 (92.3)	14 (87.5)	2 (40.0)	26 (78.8)	67 (78.8)
*BRCA2* test results, *n* (%)						
Positive	2 (11.1)	0	0	0	1 (3.0)	3 (3.5)
Negative	1 (5.6)	1 (7.7)	2 (12.5)	3 (60.0)	4 (12.1)	11 (12.9)
Unknown	1 (5.6)	0	0	0	1 (3.0)	2 (2.4)
Not tested	14 (77.8)	12 (92.3)	14 (87.5)	2 (40.0)	27 (81.8)	69 (81.2)
Median no. of prior anticancer therapies (min, max)	2.5 (1, 5)	2.0 (1, 6)	2.0 (1, 6)	3.0 (2, 5)	3.0 (1, 7)	3.0 (1, 7)

Abbreviations: ECOG PS=Eastern Cooperative Oncology Group Performance Status; i.v.=intravenous.

a Primary cancer diagnosis sites, including: appendix (*n*=2), colon (*n*=2), endometrium (*n*=2), pleura (*n*=2), stomach (*n*=2), abdomen (*n*=1), adrenal glands (*n*=1), back (*n*=1), bladder (*n*=1), oesophagus (*n*=1), ear (*n*=1), face (*n*=1), forearm (*n*=1), gastroesophageal junction (*n*=1), left leg (*n*=1), liver (*n*=2), prostate (*n*=1), shoulder (*n*=1), skin (*n*=1), testes (*n*=1), and not available (*n*=5).

**Table 2:** Treatment-emergent adverse events of any grade reported in >20% of patients in any treatment group

					**Arm A, oral rucaparib+carboplatin (*n*=33) *n* (%)**		
**Event**	**Arm A, i.v. rucaparib+carboplatin (*n*=18), *n* (%)**	**Arm B, i.v. rucaparib+carboplatin/paclitaxel (*n*=13), *n* (%)**	**Arm C, i.v. rucaparib+cisplatin/pemetrexed (*n*=16), *n* (%)**	**Arm D, i.v. rucaparib+epirubicin/ cyclophosphamide (*n*=5), *n* (%)**	**Any grade**	**Grade ⩾3**	**All patients (*N*=85), *n* (%)**
Nausea	14 (77.8)	7 (53.8)	10 (62.5)	4 (80.0)	23 (69.7)	4 (12.1)	58 (68.2)
Fatigue	14 (77.8)	4 (30.8)	14 (87.5)	1 (20.0)	20 (60.6)	2 (6.1)	53 (62.4)
Constipation	10 (55.6)	5 (38.5)	9 (56.3)	1 (20.0)	17 (51.5)	1 (3.0)	42 (49.4)
Vomiting	8 (44.4)	5 (38.5)	8 (50.0)	1 (20.0)	16 (48.5)	4 (12.1)	38 (44.7)
Anaemia	6 (33.3)	2 (15.4)	8 (50.0)	0	18 (54.5)	4 (12.1)	34 (40.0)
Neutropenia	8 (44.4)	7 (53.8)	8 (50.0)	0	11 (33.3)	7 (21.2)	34 (40.0)
Diarrhoea	7 (38.9)	5 (38.5)	9 (56.3)	1 (20.0)	11 (33.3)	1 (3.0)	33 (38.8)
Thrombocytopenia	6 (33.3)	3 (23.1)	5 (31.3)	0	15 (45.5)	9 (27.3)	29 (34.1)
Decreased appetite	5 (27.8)	0	4 (25.0)	2 (40.0)	12 (36.4)	0	23 (27.1)
Abdominal pain	3 (16.7)	3 (23.1)	1 (6.3)	0	12 (36.4)	2 (6.1)	19 (22.4)
Headache	5 (27.8)	2 (15.4)	4 (25.0)	1 (20.0)	7 (21.2)	0	19 (22.4)
Dyspnoea	5 (27.8)	1 (7.7)	5 (31.3)	1 (20.0)	7 (21.2)	2 (6.1)	19 (22.4)
Cough	2 (11.1)	0	6 (37.5)	0	8 (24.2)	0	16 (18.8)
Lethargy	1 (5.6)	6 (46.2)	1 (6.3)	0	7 (21.2)	0	15 (17.6)
Back pain	3 (16.7)	1 (7.7)	1 (6.3)	0	10 (30.3)	1 (3.0)	15 (17.6)
Alopecia	2 (11.1)	10 (76.9)	2 (12.5)	0	1 (3.0)	0	15 (17.6)
Stomatitis	4 (22.2)	6 (46.2)	1 (6.3)	1 (20.0)	2 (6.1)	0	14 (16.5)
Pyrexia	3 (16.7)	1 (7.7)	3 (18.8)	2 (40.0)	5 (15.2)	0	14 (16.5)
Dyspepsia	2 (11.1)	2 (15.4)	4 (25.0)	0	4 (12.1)	0	12 (14.1)
Oral candidiasis	1 (5.6)	2 (15.4)	6 (37.5)	1 (20.0)	1 (3.0)	0	11 (12.9)
Arthralgia	2 (11.1)	3 (23.1)	4 (25.0)	0	2 (6.1)	0	11 (12.9)
Neuropathy peripheral	0	7 (53.8)	3 (18.8)	0	0	0	10 (11.8)
Insomnia	1 (5.6)	0	5 (31.3)	0	3 (9.1)	0	9 (10.6)
Abdominal pain upper	2 (11.1)	1 (7.7)	0	2 (40.0)	3 (9.1)	1 (3.0)	8 (9.4)
Epistaxis	1 (5.6)	0	5 (31.3)	0	0	0	6 (7.1)
Tachycardia	2 (11.1)	0	4 (25.0)	0	0	0	6 (7.1)
Asthenia	0	0	0	4 (80.0)	0	0	4 (4.7)
Influenza-like illness	0	0	4 (25.0)	0	0	0	4 (4.7)

Abbreviation: i.v.=intravenous.

**Table 3:** Effect of carboplatin on oral rucaparib PK parameters and of chemotherapy on i.v. rucaparib PK parameters

	**Ratio of rucaparib PK parameters,^a^** **mean (CV%)**	
	C_max_	AUC_0–24_
Oral rucaparib		
**Carboplatin dose**		
AUC3 (*n*=15)	1.25 (46)	1.27 (37)
AUC4 (*n*=3)	0.928 (24)	1.15 (51)
AUC5 (*n*=11)	1.14 (59)	1.00 (48)
Overall (*n*=29)	1.18 (49)	1.15 (87)
	**Ratio of rucaparib PK parameters,^b^** **mean (CV%)**	
	C_max_	AUC_0–24_
**i.v. rucaparib**		
**Chemotherapy**		
Carboplatin (*n*=6)	1.03 (16)	1.02 (15)
Carboplatin+paclitaxel (*n*=6)	0.917 (16)	0.86 (8)
Cisplatin+pemetrexed (*n*=8)	0.853 (42)	0.959 (20)
Epirubicin+cyclophosphamide (*n*=5)	0.968 (29)	0.916 (34)

Abbreviations: AUC=area under the concentration time curve; AUC_0–24_=AUC for time 0 to 24 h; C_max_=maximum plasma drug concentration; CV=coefficient of variation; i.v.=intravenous; PK=pharmacokinetic.

a Ratio of rucaparib PK parameter on day 1/day −5 with single oral dose of rucaparib (80, 120, 180, 240, and 360 mg) on day −5 and single oral dose of rucaparib (80, 120, 180, 240, and 360 mg) followed 1.5 h later with 30 min i.v. infusion of carboplatin (AUC3, AUC4, or AUC5) on day 1.

b Ratio of rucaparib PK parameter on day 1/day −10 with i.v. dose of rucaparib (12, 18, or 24 mg) on day −10 and i.v. rucaparib plus i.v. chemotherapy on day 1; for AUC4, *n*=2 for AUC ratio; for overall, *n*=28 for AUC ratio.

**Table 4:** Investigator-assessed objective tumour response

**Best response^a^**	**Arm A, i.v. rucaparib+carboplatin (*n*=18), *n* (%)**	**Arm B, i.v. rucaparib+carboplatin/paclitaxel (*n*=13), *n* (%)**	**Arm C, i.v. rucaparib+cisplatin/pemetrexed (*n*=16), *n* (%)**	**Arm D, i.v. rucaparib+epirubicin/ cyclophosphamide (*n*=5), *n* (%)**	**Arm A, oral rucaparib+carboplatin (*n*=33), *n* (%)**	**All patients (*N*=85), *n* (%)**
Complete response	0	0	1 (6.3)	0	0	1 (1.2)
Partial response	3 (16.7)	1 (7.7)	2 (12.5)	0	3 (9.1)	9 (10.6)
Stable disease	9 (50.0)	8 (61.5)	7 (43.8)	1 (20.0)	18 (54.5)	43 (50.6)
Progressive disease	5 (27.8)	3 (23.1)	3 (18.8)	4 (80.0)	9 (27.3)	24 (28.2)
Missing	1 (5.6)	1 (7.7)	3 (18.8)	0	3 (9.1)	8 (9.4)

Abbreviation: i.v.=intravenous.

a According to Response Evaluation Criteria in Solid Tumor version 1.1.

## References

[bib1] Ashworth A (2008) A synthetic lethal therapeutic approach: poly(ADP) ribose polymerase inhibitors for the treatment of cancers deficient in DNA double-strand break repair. J Clin Oncol 26: 3785–3790. 18591545 10.1200/JCO.2008.16.0812

[bib2] Audeh MW, Carmichael J, Penson RT, Friedlander M, Powell B, Bell-McGuinn KM, Scott C, Weitzel JN, Oaknin A, Loman N, Lu K, Schmutzler RK, Matulonis U, Wickens M, Tutt A (2010) Oral poly(ADP-ribose) polymerase inhibitor olaparib in patients with BRCA1 or BRCA2 mutations and recurrent ovarian cancer: a proof-of-concept trial. Lancet 376: 245–251. 20609468 10.1016/S0140-6736(10)60893-8

[bib3] Bryant HE, Schultz N, Thomas HD, Parker KM, Flower D, Lopez E, Kyle S, Meuth M, Curtin NJ, Helleday T (2005) Specific killing of BRCA2-deficient tumours with inhibitors of poly(ADP-ribose) polymerase. Nature 434: 913–917. 15829966 10.1038/nature03443

[bib4] Calabrese CR, Almassy R, Barton S, Batey MA, Calvert AH, Canan-Koch S, Durkacz BW, Hostomsky Z, Kumpf RA, Kyle S, Li J, Maegley K, Newell DR, Notarianni E, Stratford IJ, Skalitzky D, Thomas HD, Wang LZ, Webber SE, Williams KJ, Curtin NJ (2004) Anticancer chemosensitization and radiosensitization by the novel poly(ADP-ribose) polymerase-1 inhibitor AG14361. J Natl Cancer Inst 96: 56–67. 14709739 10.1093/jnci/djh005

[bib5] Ceccaldi R, Liu JC, Amunugama R, Hajdu I, Primack B, Petalcorin MI, O'Connor KW, Konstantinopoulos PA, Elledge SJ, Boulton SJ, Yusufzai T, D'Andrea AD (2015) Homologous-recombination-deficient tumours are dependent on Poltheta-mediated repair. Nature 518: 258–262. 25642963 10.1038/nature14184 PMC4415602

[bib6] Clark CC, Weitzel JN, O'Connor TR (2012) Enhancement of synthetic lethality via combinations of ABT-888, a PARP inhibitor, and carboplatin *in vitro* and *in vivo* using BRCA1 and BRCA2 isogenic models. Mol Cancer Ther 11: 1948–1958. 22778154 10.1158/1535-7163.MCT-11-0597 PMC3551628

[bib7] Dent RA, Lindeman GJ, Clemons M, Wildiers H, Chan A, McCarthy NJ, Singer CF, Lowe ES, Watkins CL, Carmichael J (2013) Phase I trial of the oral PARP inhibitor olaparib in combination with paclitaxel for first- or second-line treatment of patients with metastatic triple-negative breast cancer. Breast Cancer Res 15: R88. 24063698 10.1186/bcr3484 PMC3979135

[bib8] Donawho CK, Luo Y, Luo Y, Penning TD, Bauch JL, Bouska JJ, Bontcheva-Diaz VD, Cox BF, DeWeese TL, Dillehay LE, Ferguson DC, Ghoreishi-Haack NS, Grimm DR, Guan R, Han EK, Holley-Shanks RR, Hristov B, Idler KB, Jarvis K, Johnson EF, Kleinberg LR, Klinghofer V, Lasko LM, Liu X, Marsh KC, McGonigal TP, Meulbroek JA, Olson AM, Palma JP, Rodriguez LE, Shi Y, Stavropoulos JA, Tsurutani AC, Zhu GD, Rosenberg SH, Giranda VL, Frost DJ (2007) ABT-888, an orally active poly(ADP-ribose) polymerase inhibitor that potentiates DNA-damaging agents in preclinical tumor models. Clin Cancer Res 13: 2728–2737. 17473206 10.1158/1078-0432.CCR-06-3039

[bib9] Drew Y, Ledermann J, Hall G, Rea D, Glasspool R, Highley MS, Jayson GC, Sludden J, Murray J, Jamieson D, Halford S, Acton G, Backholer Z, Mangano R, Boddy A, Curtin N, Plummer E (2016) Phase 2 multicentre trial investigating intermittent and continuous dosing schedules of the poly(ADP-ribose) polymerase inhibitor rucaparib in germline BRCA mutation carriers with advanced ovarian and breast cancer. Br J Cancer 114: 723–730. 27002934 10.1038/bjc.2016.41 PMC4882768

[bib10] Drew Y, Mulligan EA, Vong WT, Thomas HD, Kahn S, Kyle S, Mukhopadhyay A, Los G, Hostomsky Z, Plummer ER, Edmondson RJ, Curtin NJ (2011) Therapeutic potential of poly(ADP-ribose) polymerase inhibitor AG014699 in human cancers with mutated or methylated BRCA1 or BRCA2. J Natl Cancer Inst 103: 334–346. 21183737 10.1093/jnci/djq509

[bib11] Eisenhauer EA, Therasse P, Bogaerts J, Schwartz LH, Sargent D, Ford R, Dancey J, Arbuck S, Gwyther S, Mooney M, Rubinstein L, Shankar L, Dodd L, Kaplan R, Lacombe D, Verweij J (2009) New response evaluation criteria in solid tumours: revised RECIST guideline (version 1.1). Eur J Cancer 45: 228–247. 19097774 10.1016/j.ejca.2008.10.026

[bib12] Evers B, Drost R, Schut E, de Bruin M, van der Burg E, Derksen PWB, Holstege H, Liu X, van Drunen E, Beverloo HB, Smith GCM, Martin NMB, Lau A, O'Connor MJ, Jonkers J (2008) Selective inhibition of BRCA2-deficient mammary tumor cell growth by AZD2281 and cisplatin. Clin Cancer Res 14: 3916–3925. 18559613 10.1158/1078-0432.CCR-07-4953

[bib13] Farmer H, McCabe N, Lord CJ, Tutt ANJ, Johnson DA, Richardson TB, Santarosa M, Dillon KJ, Hickson I, Knights C, Martin NMB, Jackson SP, Smith GCM, Ashworth A (2005) Targeting the DNA repair defect in BRCA mutant cells as a therapeutic strategy. Nature 434: 917–921. 15829967 10.1038/nature03445

[bib14] Fast DM, Kelley M, Viswanathan CT, O'Shaughnessy J, King SP, Chaudhary A, Weiner R, DeStefano AJ, Tang D (2009) Workshop report and follow-up—AAPS workshop on current topics in GLP bioanalysis: assay reproducibility for incurred samples—implications of Crystal City recommendations. AAPS J 11: 238–241. 19381839 10.1208/s12248-009-9100-9 PMC2691460

[bib15] Fong PC, Boss DS, Yap TA, Tutt A, Wu P, Mergui-Roelvink M, Mortimer P, Swaisland H, Lau A, O'Connor MJ, Ashworth A, Carmichael J, Kaye SB, Schellens JHM, de Bono JS (2009) Inhibition of poly(ADP-ribose) polymerase in tumors from BRCA mutation carriers. N Engl J Med 361: 123–134. 19553641 10.1056/NEJMoa0900212

[bib16] Helleday T (2011) The underlying mechanism for the PARP and BRCA synthetic lethality: clearing up the misunderstandings. Mol Oncol 5: 387–393. 21821475 10.1016/j.molonc.2011.07.001 PMC5528309

[bib17] Helleday T, Lo J, van Gent DC, Engelward BP (2007) DNA double-strand break repair: from mechanistic understanding to cancer treatment. DNA Repair 6: 923–935. 17363343 10.1016/j.dnarep.2007.02.006

[bib18] Helleday T, Petermann E, Lundin C, Hodgson B, Sharma RA (2008) DNA repair pathways as targets for cancer therapy. Nat Rev Cancer 8: 193–204. 18256616 10.1038/nrc2342

[bib19] Ihnen M, zu Eulenburg C, Kolarova T, Qi JW, Manivong K, Chalukya M, Dering J, Anderson L, Ginther C, Meuter A, Winterhoff B, Jones S, Velculescu VE, Venkatesan N, Rong H-M, Dandekar S, Udar N, Jänicke F, Los G, Slamon DJ, Konecny GE (2013) Therapeutic potential of the poly(ADP-ribose) polymerase inhibitor rucaparib for the treatment of sporadic human ovarian cancer. Mol Cancer Ther 12: 1002–1015. 23729402 10.1158/1535-7163.MCT-12-0813 PMC3963026

[bib20] Kaufman B, Shapira-Frommer R, Schmutzler RK, Audeh MW, Friedlander M, Balmaña J, Mitchell G, Fried G, Stemmer SM, Hubert A, Rosengarten O, Steiner M, Loman N, Bowen K, Fielding A, Domchek SM (2015) Olaparib monotherapy in patients with advanced cancer and a germline BRCA1/2 mutation. J Clin Oncol 33: 244–250. 25366685 10.1200/JCO.2014.56.2728 PMC6057749

[bib21] Kaye SB, Lubinski J, Matulonis U, Ang JE, Gourley C, Karlan BY, Amnon A, Bell-McGuinn KM, Chen LM, Friedlander M, Safra T, Vergote I, Wickens M, Lowe ES, Carmichael J, Kaufman B (2012) Phase II, open-label, randomized, multicenter study comparing the efficacy and safety of olaparib, a poly (ADP-ribose) polymerase inhibitor, and pegylated liposomal doxorubicin in patients with BRCA1 or BRCA2 mutations and recurrent ovarian cancer. J Clin Oncol 30: 372–379. 22203755 10.1200/JCO.2011.36.9215

[bib22] Khan OA, Gore M, Lorigan P, Stone J, Greystoke A, Burke W, Carmichael J, Watson AJ, McGown G, Thorncroft M, Margison GP, Califano R, Larkin J, Wellman S, Middleton MR (2011) A phase I study of the safety and tolerability of olaparib (AZD2281, KU0059436) and dacarbazine in patients with advanced solid tumours. Br J Cancer 104: 750–755. 21326243 10.1038/bjc.2011.8 PMC3048218

[bib23] Konstantinopoulos PA, Ceccaldi R, Shapiro GI, D'Andrea AD (2015) Homologous recombination deficiency: exploiting the fundamental vulnerability of ovarian cancer. Cancer Discov 5: 1137–1154. 26463832 10.1158/2159-8290.CD-15-0714 PMC4631624

[bib24] Kristeleit RS, Burris HA, LoRusso P, Patel MR, Asghar US, El-Khouly F, Calvert AH, Infante JR, Hilton JF, Tolaney SM, Kittaneh M, Giordano H, Borrow J, Jaw-Tsai SS, Shapiro G (2014) Phase 1/2 study of oral rucaparib: final phase 1 results. J Clin Oncol 32: 2573.

[bib25] Lee JM, Hays JL, Annunziata CM, Noonan AM, Minasian L, Zujewski JA, Yu M, Gordon N, Ji J, Sissung TM, Figg WD, Azad N, Wood BJ, Doroshow J, Kohn EC (2014a) Phase I/Ib study of olaparib and carboplatin in BRCA1 or BRCA2 mutation-associated breast or ovarian cancer with biomarker analyses. J Natl Cancer Inst 106: dju089. 24842883 10.1093/jnci/dju089 PMC4049120

[bib26] Lee JM, Ledermann JA, Kohn EC (2014b) PARP Inhibitors for BRCA1/2 mutation-associated and BRCA-like malignancies. Ann Oncol 25: 32–40. 24225019 10.1093/annonc/mdt384 PMC3868320

[bib27] Mateos-Gomez PA, Gong F, Nair N, Miller KM, Lazzerini-Denchi E, Sfeir A (2015) Mammalian polymerase [THGR] promotes alternative NHEJ and suppresses recombination. Nature 518: 254–257. 25642960 10.1038/nature14157 PMC4718306

[bib28] Moynahan ME, Chiu JW, Koller BH, Jasin M (1999) Brca1 controls homology-directed DNA repair. Mol Cell 4: 511–518. 10549283 10.1016/s1097-2765(00)80202-6

[bib29] Moynahan ME, Pierce AJ, Jasin M (2001) BRCA2 is required for homology-directed repair of chromosomal breaks. Mol Cell 7: 263–272. 11239455 10.1016/s1097-2765(01)00174-5

[bib30] Murai J, Huang SY, Das BB, Renaud A, Zhang Y, Doroshow JH, Ji J, Takeda S, Pommier Y (2012) Trapping of PARP1 and PARP2 by clinical PARP inhibitors. Cancer Res 72: 5588–5599. 23118055 10.1158/0008-5472.CAN-12-2753 PMC3528345

[bib31] O'Connor MJ (2015) Targeting the DNA damage response in cancer. Mol Cell 60: 547–560. 26590714 10.1016/j.molcel.2015.10.040

[bib32] Pedersen B, Konstantinopoulos PA, Spillman MA, De S (2013) Copy neutral loss of heterozygosity is more frequent in older ovarian cancer patients. Genes Chromosomes Cancer 52: 794–801. 23716468 10.1002/gcc.22075 PMC3767172

[bib33] Plummer R, Jones C, Middleton M, Wilson R, Evans J, Olsen A, Curtin N, Boddy A, McHugh P, Newell D, Harris A, Johnson P, Steinfeldt H, Dewji R, Wang D, Robson L, Calvert H (2008) Phase I study of the poly(ADP-ribose) polymerase inhibitor, AG014699, in combination with temozolomide in patients with advanced solid tumors. Clin Cancer Res 14: 7917–7923. 19047122 10.1158/1078-0432.CCR-08-1223 PMC2652879

[bib34] Plummer R, Lorigan P, Steven N, Scott L, Middleton MR, Wilson RH, Mulligan E, Curtin N, Wang D, Dewji R, Abbattista A, Gallo J, Calvert H (2013) A phase II study of the potent PARP inhibitor, Rucaparib (PF-01367338, AG014699), with temozolomide in patients with metastatic melanoma demonstrating evidence of chemopotentiation. Cancer Chemother Pharmacol 71: 1191–1199. 23423489 10.1007/s00280-013-2113-1

[bib35] Plummer R, Stephens P, Aissat-Daudigny L, Cambois A, Moachon G, Brown PD, Campone M (2014) Phase 1 dose-escalation study of the PARP inhibitor CEP-9722 as monotherapy or in combination with temozolomide in patients with solid tumors. Cancer Chemother Pharmacol 74: 257–265. 24880570 10.1007/s00280-014-2486-9 PMC4112042

[bib36] Rajan A, Carter CA, Kelly RJ, Gutierrez M, Kummar S, Szabo E, Yancey MA, Ji J, Mannargudi B, Woo S, Spencer S, Figg WD, Giaccone G (2012) A phase I combination study of olaparib with cisplatin and gemcitabine in adults with solid tumors. Clin Cancer Res 18: 2344–2351. 22371451 10.1158/1078-0432.CCR-11-2425 PMC6368967

[bib37] Samol J, Ranson M, Scott E, Macpherson E, Carmichael J, Thomas A, Cassidy J (2012) Safety and tolerability of the poly(ADP-ribose) polymerase (PARP) inhibitor, olaparib (AZD2281) in combination with topotecan for the treatment of patients with advanced solid tumors: a phase I study. Invest New Drugs 30: 1493–1500. 21590367 10.1007/s10637-011-9682-9

[bib38] Sandhu SK, Schelman WR, Wilding G, Moreno V, Baird RD, Miranda S, Hylands L, Riisnaes R, Forster M, Omlin A, Kreischer N, Thway K, Gevensleben H, Sun L, Loughney J, Chatterjee M, Toniatti C, Carpenter CL, Iannone R, Kaye SB, de Bono JS, Wenham RM (2013) The poly(ADP-ribose) polymerase inhibitor niraparib (MK4827) in BRCA mutation carriers and patients with sporadic cancer: a phase 1 dose-escalation trial. Lancet Oncol 14: 882–892. 23810788 10.1016/S1470-2045(13)70240-7

[bib39] Schreiber V, Dantzer F, Ame JC, de Murcia G (2006) Poly(ADP-ribose): novel functions for an old molecule. Nat Rev Mol Cell Biol 7: 517–528. 16829982 10.1038/nrm1963

[bib40] Shapira-Frommer R, Oza AM, Domchek SM, Balmana J, Patel MR, Chen L-M, Drew Y, Burris HA, Korach J, Flynn M, Bowering VL, Morgan MA, Watkins SP, Simpson D, Goble S, Maloney L, Kristeleit RS (2015) A phase 2 open-label, multicenter study of single-agent rucaparib in the treatment of patients with relapsed ovarian cancer and a deleterious BRCA mutation. J Clin Oncol 33: 5513.

[bib41] Swisher EM, Lin KK, Oza AM, Scott CL, Giordano H, Sun J, Konecny GE, Coleman RL, Tinker AV, O'Malley DM, Kristeleit RS, Ma L, Bell-McGuinn KM, Brenton JD, Cragun JM, Oaknin A, Ray-Coquard I, Harrell MI, Mann E, Kaufmann SH, Floquet A, Leary A, Harding TC, Goble S, Maloney L, Isaacson J, Allen AR, Rolfe L, Yelensky R, Raponi M, McNeish IA (2017) Rucaparib in relapsed, platinum-sensitive high-grade ovarian carcinoma (ARIEL2 Part 1): an international, multicentre, open-label, phase 2 trial. Lancet Oncol 18: 75–87. 27908594 10.1016/S1470-2045(16)30559-9

[bib42] Thomas HD, Calabrese CR, Batey MA, Canan S, Hostomsky Z, Kyle S, Maegley KA, Newell DR, Skalitzky D, Wang LZ, Webber SE, Curtin NJ (2007) Preclinical selection of a novel poly(ADP-ribose) polymerase inhibitor for clinical trial. Mol Cancer Ther 6: 945–956. 17363489 10.1158/1535-7163.MCT-06-0552

[bib43] Tutt A, Robson M, Garber JE, Domchek SM, Audeh MW, Weitzel JN, Friedlander M, Arun B, Loman N, Schmutzler RK, Wardley A, Mitchell G, Earl H, Wickens M, Carmichael J (2010) Oral poly(ADP-ribose) polymerase inhibitor olaparib in patients with BRCA1 or BRCA2 mutations and advanced breast cancer: a proof-of-concept trial. Lancet 376: 235–244. 20609467 10.1016/S0140-6736(10)60892-6

[bib44] US Food and Drug Administration (2001) Bioanalytical Method Validation (May 2001). Available from http://www.fda.gov/Drugs/GuidanceComplianceRegulatoryInformation/Guidances/ucm064964.htm.

[bib45] Venkitaraman AR (2002) Cancer susceptibility and the functions of BRCA1 and BRCA2. Cell 108: 171–182. 11832208 10.1016/s0092-8674(02)00615-3

[bib46] Viswanathan CT, Bansal S, Booth B, DeStefano AJ, Rose MJ, Sailstad J, Shah VP, Skelly JP, Swann PG, Weiner R (2007) Workshop/conference report—quantitative bioanalytical methods validation and implementation: best practices for chromatographic and ligand binding assays. AAPS J 9: E30–E42. 10.1007/s11095-007-9291-7 17458684

