# Supplementary material for: A phase I study of intravenous and oral rucaparib in combination with chemotherapy in patients with advanced solid tumours
Source: Br J Cancer. 2017 Feb 21;116(7):884–92. doi: 10.1038/bjc.2017.36 (PMC5379148; doi:10.1038/bjc.2017.36)
Supplement: Supplementary Information [file bjc201736x1.docx]

# Supplementary Appendix

## Supplementary Methods

## Pharmacokinetics (PK). Plasma PK parameters for rucaparib were estimated using noncompartmental methods and included: maximum plasma drug concentration (C_max_); time to maximum plasma concentration (t_max_); area under the plasma concentration time curve from time 0 to the last sampling time with measurable values (AUC_0-t_), from time 0 to 24 hours (AUC_0-24_) and from time 0 to infinity (AUC_inf_); and half-life (t_1/2_). The oral bioavailability of rucaparib was calculated as the ratio of dose-normalised AUC_0-t_ or AUC_inf_ (data permitting) determined using oral rucaparib PK data collected on cycle 1 day –5 to that determined using the intravenous PK data collected on cycle 1 day –10. The t_max_ was summarised with medians and ranges. The accumulation ratio after multiple oral rucaparib dosing (once daily) was assessed as the ratio of the AUC_0-24_ determined using PK collected on cycle 1 day 14 to the AUC_0-24_ after the cycle 1 day –5 lead-in dose. The ratio of the C_max_ on day 14 to the C_max_ on day –5 was also calculated. Additionally, the ratio of the AUC_0-24_ on cycle 1 day 14 to the AUC_inf_ after the cycle 1 day –5 lead-in dose was calculated as an indication for any time-dependent change in rucaparib PK.

## Table S1. Treatment-related adverse events of any grade reported in >20% of patients in any treatment group

| Adverse event | Arm A  i.v. rucaparib + carboplatin  (*n* = 18)  *n* (%) | Arm B  i.v. rucaparib + carboplatin/paclitaxel  (*n* = 13)  *n* (%) | Arm C  i.v. rucaparib + cisplatin/pemetrexed  (*n* = 16)  *n* (%) | Arm D  i.v. rucaparib + epirubicin/ cyclophosphamide  (*n* = 5)  *n* (%) | Arm A  Oral rucaparib + carboplatin  (*n* = 33)  *n* (%) | All patients  (*N* = 85)  *n* (%) |
| --- | --- | --- | --- | --- | --- | --- |
| ≥1 treatment-related adverse event | 18 (100.0) | 13 (100.0) | 15 (93.8) | 4 (80.0) | 30 (90.9) | 80 (94.1) |
| Nausea | 12 (66.7) | 6 (46.2) | 10 (62.5) | 3 (60.0) | 17 (51.5) | 48 (56.5) |
| Fatigue | 10 (55.6) | 3 (23.1) | 13 (81.3) | 0 | 19 (57.6) | 45 (52.9) |
| Neutropenia | 7 (38.9) | 7 (53.8) | 8 (50.0) | 0 | 10 (30.3) | 32 (37.6) |
| Anaemia | 5 (27.8) | 2 (15.4) | 8 (50.0) | 0 | 16 (48.5) | 31 (36.5) |
| Vomiting | 7 (38.9) | 4 (30.8) | 8 (50.0) | 1 (20.0) | 8 (24.2) | 28 (32.9) |
| Thrombocytopenia | 5 (27.8) | 3 (23.1) | 5 (31.3) | 0 | 14 (42.4) | 27 (31.8) |
| Constipation | 2 (11.1) | 5 (38.5) | 5 (31.3) | 0 | 10 (30.3) | 22 (25.9) |
| Diarrhoea | 1 (5.6) | 4 (30.8) | 5 (31.3) | 0 | 7 (21.2) | 17 (20.0) |
| Decreased appetite | 1 (5.6) | 0 | 4 (25.0) | 2 (40.0) | 10 (30.3) | 17 (20.0) |
| Lethargy | 1 (5.6) | 6 (46.2) | 1 (6.3) | 0 | 7 (21.2) | 15 (17.6) |
| Alopecia | 2 (11.1) | 10 (76.9) | 2 (12.5) | 0 | 1 (3.0) | 15 (17.6) |
| Stomatitis | 4 (22.2) | 6 (46.2) | 1 (6.3) | 1 (20.0) | 2 (6.1) | 14 (16.5) |
| Neuropathy peripheral | 0 | 7 (53.8) | 3 (18.8) | 0 | 0 | 10 (11.8) |
| Leukopenia | 2 (11.1) | 3 (23.1) | 1 (6.3) | 0 | 1 (3.0) | 7 (8.2) |
| Myalgia | 1 (5.6) | 3 (23.1) | 0 | 1 (20.0) | 2 (6.1) | 7 (8.2) |
| Lymphopenia | 4 (22.2) | 0 | 1 (6.3) | 0 | 1 (3.0) | 6 (7.1) |
| Asthenia | 0 | 0 | 0 | 3 (60.0) | 0 | 3 (3.5) |

Abbreviation: i.v. = intravenous.

## Table S2. Summary of PK parameters for rucaparib following single i.v. doses on day –10

| PK variable^b^ | Rucaparib dose^a^ | | | | |
| --- | --- | --- | --- | --- | --- |
|  | 12 mg  (*n* = 11) | 18 mg  (*n* = 10) | 24 mg  (*n* = 7) | 27 mg  (*n* = 4) | 40 mg  (*n* = 3) |
| t_max_, h | 0.50 (0.25, 0.50) | 0.50 (0.25, 0.50) | 0.50 (0.25, 0.50) | 0.38 (0.25, 0.50) | 0.50 (0.50, 0.50) |
| C_max_, ng mL^–1^ | 324 (29.7) | 498 (33.1) | 627 (43.1) | 866 (14.6) | 1610 (46.5) |
| AUC_0-24_, ng×h mL^–1^ | 549 (28.4) | 812 (59.3) | 1220 (58.7) | 1450 (23.8) | 2530 (41.9) |
| AUC_inf_, ng×h mL^–1^ | 740 (23.3) | 976 (74.4) | 1710 (64.3) | 1900 (38.8) | 2880 (44.4) |
| CL, L h^–1^ | 16.2 (23.3) | 18.4 (74.4) | 14.0 (64.3) | 14.2 (38.8) | 13.9 (44.5) |
| V_ss_, L | 258 (48.5) | 234 (32.7) | 262 (61.2) | 191 (31.6) | 113 (30.8) |
| t_1/2_, h | 18.70 (32.1) | 16.36 (22.1) | 20.72 (28.1) | 16.81 (36.9) | 11.86 (18.7) |

Abbreviations: AUC_0-24_ = area under the concentration time curve for time 0 to 24 hours; AUC_inf_ = area under the concentration time curve for time 0 to infinity; CL = clearance; C_max_ = maximum plasma drug concentration; CV = coefficient of variation; i.v. = intravenous; PK = pharmacokinetic; t_1/2_ = half-life; t_max_ = time to maximum plasma concentration; V_ss_ = steady state volume of distribution.

^a^At 12 mg, *n* = 10 for AUC_inf_, CL, V_ss_, and t_1/2_, and at 18 mg, *n* = 9 for AUC_inf_, CL, V_ss_, and t_1/2_.

^b^t_max_ is presented as median (minimum, maximum), t_1/2_ is presented as arithmetic mean (CV%), and all other parameters are presented as geometric mean (CV%).

## Table S3. Summary of PK parameters for rucaparib following single oral doses on day –5

| PK variable^b^ | Rucaparib dose^a^ | | | | | | | | |
| --- | --- | --- | --- | --- | --- | --- | --- | --- | --- |
|  | 12 mg  (*n* = 3) | 36 mg  (*n* = 8) | 48 mg  (*n* = 8) | 72 mg  (*n* = 6) | 80 mg  (*n* = 4) | 120 mg  (*n* = 4) | 180 mg  (*n* = 3) | 240 mg  (*n* = 9) | 360 mg  (*n* = 11) |
| t_max_, h | 1.00  (0.50, 2.50) | 2.00  (0.00, 4.00) | 1.25  (1.00, 4.00) | 2.00  (0.50, 6.00) | 2.50  (2.50, 6.00) | 4.00  (2.50, 6.00) | 4.00  (1.50, 6.00) | 4.00  (1.00, 6.00) | 4.00  (1.00, 10.00) |
| C_max_, ng mL^–1^ | 24  (28.3) | 90  (52.3) | 80  (104.0) | 108  (50.5) | 114  (83.6) | 274  (32.6) | 410  (52.5) | 442  (58.7) | 814  (42.3) |
| AUC_0-24_, ng×h mL^–1^ | 155  (30.6) | 696  (55.6) | 620  (146) | 893  (53.7) | 1270  (68.4) | 2920  (36.7) | 5610  (38) | 5030  (62) | 9170  (62.1) |
| AUC_inf_,  ng×h mL^–1^ | 225  (39.7) | 1100  (41.9) | 922  (150) | 1090  (49.4) | 1920  (132) | 4070  (46.6) | 8410  (68.5) | 6990  (55.9) | 11000  (46.9) |
| CL/F, L h^–1^ | 53.4  (39.7) | 32.7  (41.9) | 52.1  (150) | 65.9  (49.4) | 41.6  (132) | 29.5  (46.6) | 21.4  (68.6) | 34.4  (56.1) | 32.8  (46.8) |
| t_1/2_, h | 18.65  (8.39) | 18.01  (29.63) | 20.34 (27.28) | 25.09 (56.48) | 20.15  (48.62) | 13.26 (16.19) | 19.23 (16.84) | 17.69 (33.28) | 18.25 (35.78) |
| F, % | 33.4  (13.8) | 45.3  (37.8) | 33.6  (53.5) | 30.1  (33.3) | 30.7  (52.4) | 41.1  (26.1) | - | - | - |

Abbreviations: AUC_0-24_ = area under the concentration time curve for time 0 to 24 hours; AUC_inf_ = area under the concentration time curve for time 0 to infinity; CL/F = apparent plasma clearance of a drug after oral administration; C_max_ = maximum plasma drug concentration; CV = coefficient of variation; F = oral bioavailability [(AUC_PO, day -5_/AUC_i.v., day -10_)×(D_i.v._/D_PO_)]; PK = pharmacokinetic; t_1/2_ = half-life; t_max_ = time to maximum plasma concentration.

^a^At 36 mg, *n* = 7 for AUC_inf_, CL/F; at 48 mg, *n* = 6 for AUC_inf_, CL/F; at 72 mg, *n* = 3 for AUC_inf_, CL/F; at 80 mg, *n* = 2 for AUC_inf_, CL/F; at 180 mg, *n* = 2 for AUC_inf_, CL/F.

^b^t_max_ is presented as median (minimum, maximum), t_1/2_ is presented as arithmetic mean (CV%), and all other parameters are presented as geometric mean (CV%).

## Table S4. Summary of PK parameters for rucaparib following single oral doses on day 1

| PK variable^b^ | Rucaparib dose^a^ | | | | |
| --- | --- | --- | --- | --- | --- |
|  | 80 mg  (*n* = 3) | 120 mg  (*n* = 3) | 180 mg  (*n* = 3) | 240 mg  (*n* = 9) | 360 mg  (*n* = 11) |
| t_max_, h | 4.00 (4.00, 6.00) | 4.00 (4.00, 6.00) | 6.00 (4.00, 6.00) | 4.00 (1.50, 10.00) | 4.00 (1.50, 10.00) |
| C_max,_ ng mL^–1^ | 99 (30) | 344 (47.1) | 527 (95.5) | 493 (65) | 697 (42.2) |
| AUC_0-24_, ng×h mL^–1^ | 1380 (3.41) | 3260 (58.2) | 5920 (70.4) | 5130 (86.8) | 8280 (36.3) |
| AUC_inf_, ng×h mL^–1^ | 1700 (-) | 3770 (102) | NR | 4370 (209) | 9980 (20.5) |
| CL/F, L h^–1^ | 47.2 (-) | 37.5 (88.7) | NR | 57.1 (227) | 36.1 (20.5) |
| t_1/2_, h | 11.57 (26.40) | 8.70 (32.22) | 11.20 (-) | 11.21 (25.27) | 9.72 (35.43) |

Abbreviations: AUC_0-24_ = area under the concentration time curve for time 0 to 24 hours; AUC_inf_ = area under the concentration time curve for time 0 to infinity; CL/F = apparent plasma clearance of a drug after oral administration; C_max_ = maximum plasma drug concentration; CV = coefficient of variation; PK = pharmacokinetic; t_1/2_ = half-life; t_max_ = time to maximum plasma concentration; NR = not reportable.

^a^At 180 mg, *n* = 1 for t_1/2_.

^b^t_max_ is presented as median (minimum, maximum), t_1/2_ is presented as arithmetic mean (CV%), and all other parameters are presented as geometric mean (CV%).

## Table S5. Summary of PK parameters for rucaparib following oral q.d. doses for 14 days

| PK variable^b^ | Rucaparib dose^a^ | | | | |
| --- | --- | --- | --- | --- | --- |
|  | 80 mg  (*n* = 1) | 120 mg  (*n* = 3) | 180 mg  (*n* = 3) | 240 mg  (*n* = 8) | 360 mg  (*n* = 11) |
| t_max_, h | 2.50 (2.50, 2.50) | 2.5 (1.50, 4.00) | 4.00 (4.00, 6.00) | 4.00 (1.50, 6.00) | 4.00 (2.50, 6.00) |
| C_max_, ng mL^–1^ | 190 (-) | 375 (106) | 454 (32.6) | 527 (47.6) | 1050 (39.3) |
| AUC_0-24_, ng×h mL^–1^ | 2210 (-) | 4040 (111) | 7660 (39.2) | 7560 (61.6) | 15600 (47.4) |
| t_1/2_, h | 10.23 (-) | 12.64 (64.25) | 19.60 (-) | 17.07 (35.43) | 16.04 (32.39) |
| AR | 1.19 (-) | 1.49 (104) | 1.36 (8.48) | 1.64 (28.2) | 1.7 (46.3) |
| AR_cm_ | 1.50 (-) | 1.41 (99.8) | 1.11 (31.1) | 1.30 (29.7) | 1.29 (46.1) |
| AUC ratio [time-dependent linearity] | 0.655 (-) | 1.14 (101) | 0.785 (13.3) | 1.01 (31.6) | 1.06 (53.1) |

Abbreviations: AR = accumulation ratio [AUC_0-24, Day 14_/AUC_0-24, Day −5_]; AR_cm_ = accumulation ratio of C_max_ on day 14 to C_max_ on day –5; AUC = area under the concentration time curve; AUC_0-24_ = area under the concentration time curve for time 0 to 24 hours; C_max_ = maximum plasma drug concentration; CV = coefficient of variation; PK = pharmacokinetic; q.d. = once daily; t_1/2_ = half-life; t_max_ = time to maximum plasma concentration.

^a^At 80 mg and 180 mg, *n* = 1 for t_1/2_; at 240 mg, *n* = 7 for t_1/2_; at 360 mg, *n* = 9 for t_1/2_.

^b^t_max_ is presented as median (minimum, maximum), t_1/2_ is presented as arithmetic mean (CV%), and all other parameters are presented as geometric mean (CV%).
